# Laminin α2-Mediated Focal Adhesion Kinase Activation Triggers Alport Glomerular Pathogenesis

**DOI:** 10.1371/journal.pone.0099083

**Published:** 2014-06-10

**Authors:** Duane Delimont, Brianna M. Dufek, Daniel T. Meehan, Marisa Zallocchi, Michael Anne Gratton, Grady Phillips, Dominic Cosgrove

**Affiliations:** 1 Department of Genetics, Boys Town National Research Hospital, Omaha, Nebraska, United States of America; 2 Department of Biochemistry, University of Nebraska Medical Center, Omaha, Nebraska, United States of America; 3 Department of Otolaryngology, Saint Louis University, Saint Louis, Missouri, United States of America; Cedars-Sinai Medical Center; UCLA School of Medicine, United States of America

## Abstract

It has been known for some time that laminins containing α1 and α2 chains, which are normally restricted to the mesangial matrix, accumulate in the glomerular basement membranes (GBM) of Alport mice, dogs, and humans. We show that laminins containing the α2 chain, but not those containing the α1 chain activates focal adhesion kinase (FAK) on glomerular podocytes *in vitro* and *in vivo*. CD151-null mice, which have weakened podocyte adhesion to the GBM rendering these mice more susceptible to biomechanical strain in the glomerulus, also show progressive accumulation of α2 laminins in the GBM, and podocyte FAK activation. Analysis of glomerular mRNA from both models demonstrates significant induction of MMP-9, MMP-10, MMP-12, MMPs linked to GBM destruction in Alport disease models, as well as the pro-inflammatory cytokine IL-6. SiRNA knockdown of FAK in cultured podocytes significantly reduced expression of MMP-9, MMP-10 and IL-6, but not MMP-12. Treatment of Alport mice with TAE226, a small molecule inhibitor of FAK activation, ameliorated fibrosis and glomerulosclerosis, significantly reduced proteinuria and blood urea nitrogen levels, and partially restored GBM ultrastructure. Glomerular expression of MMP-9, MMP-10 and MMP-12 mRNAs was significantly reduced in TAE226 treated animals. Collectively, this work identifies laminin α2-mediated FAK activation in podocytes as an important early event in Alport glomerular pathogenesis and suggests that FAK inhibitors, if safe formulations can be developed, might be employed as a novel therapeutic approach for treating Alport renal disease in its early stages.

## Introduction

The pathologic glomerular basement membrane in Alport syndrome is irregularly thickened and thinned, with a multilaminar or “basketweave” appearance that is unique to the disease and a definitive diagnostic test for Alport syndrome [1]. It has been shown that the thickened regions are more permeable to injected ferritin than the non-thickened regions of the GBM [2]. This property is consistent with a partially degraded matrix network, suggesting proteolytic damage may contribute to focal thickening of the Alport GBM. The type IV collagen network in Alport GBM is comprised entirely of α1(IV)/α2(IV) chains, which contains fewer interchain crosslinks than the subepithelial α3(IV)/α4(IV)/α5(IV) network found in wild type GBM [3], and is more susceptible to proteolytic degradation by endogenously expressed matrix metalloproteinases [4], [5].

Work on Alport renal disease thus far has focused on events that occur after glomerular disease is well established. The work includes roles for pro-inflammatory cytokines, such as TGF-β1 [5], [6], CTGF [7], and the adhesion molecule α1β1 integrin [8], [9], all of which contribute to the glomerular pathology in Alport syndrome. MMPs are also induced as a function of disease progression, and several MMPs, including MMP-2, MMP-9, and MMP-12 have been functionally linked to progressive destruction of the GBM [4], [5].

An unusual characteristic of Alport glomerular disease progression is the early and progressive deposition of abnormal laminins containing α1 and α2 chains in the GBM. While this phenomenon was first described many years ago [8], [10], the functional significance of this observation as it relates to molecular pathology in the glomerulus has remained unclear. Recently our laboratory demonstrated that laminin α2 in the GBM is deposited by invading mesangial cell processes [11], a process that may be triggered by biomechanical strain on the capillary tuft owing to the altered type IV collagen composition of the GBM. In this current work we identify FAK activation in podocyte foot processes specifically in regions of the GBM where abnormal laminin deposition is occurring. We observe this as early as P10, long before detectable proteinuria for Alport mice on the 129 Sv/J background, which is detectable at about 3 weeks of age [8]. We link FAK activation to elevated expression of MMP-9, MMP-10, MMP-12, and IL-6, all of which are implicated in the progressive GBM destruction associated with Alport glomerular disease. The glomerular capillary filtration barrier is comprised of fenestrated endothelium, the GBM, and the adhesive interface of the podocyte foot processes, and the slit diaphragms. Compromising any of these components would be expected to negatively impact the structural integrity of the capillary tufts, making them more susceptible to biomechanical stresses given the high hydraulic pressure of the glomerulus. We demonstrate that CD151 knockout mouse, which has a specific defect in α3β1 integrin binding affinity, a characteristic likely to impact the structural integrity of the capillary tuft [12], also shows mesangial filopodial invasion of the glomerular capillaries, deposition of α2 containing laminins in the GBM, podocyte FAK activation and MMP dysregulation. Importantly, the CD151 knockout mouse has a normal type IV collagen network in the GBM, which suggests that these events are likely due to biomechanical strain, rather than altered signaling resulting from the altered type IV collagen basement membrane composition in Alport GBM.

## Materials and Methods

### Animals

Alport mice were either autosomal recessive (COL4A3 mutant on the 129 Sv/J background [13]. CD151 knockout mice were on the FVB background and were a gift from Martin Hemler, Harvard Medical School [14]. Laminin dy/dy mice were obtained from the Jackson Laboratories (strain # 129P1/ReJ-*Lama2^dy^*/J, stock #000641). Age/strain matched wild type mice were used as controls. All animal work was done under an IACUC protocol approved by the BTNRH IACUC committee and in accordance with the USDA and NIH guidelines for the care and use of animals for research. Every effort was made to minimize stress and discomfort.

### Antibodies and inhibitors

Anti-α-actinin-4 was from Santa Cruz Biotechnology, Inc (Dallas, TX, USA, Cat #: SC-49333); anti-CD11b was from CedarLane Laboratories Limited (Hornby, Ontario, Canada, Cat #: CL8941AP); anti-Fibronectin was from Sigma (St. Louis, MO, USA, Cat #: F3648); anti-Integrin α8 was from R&D Systems (Minneapolis, MN, USA, Cat #: AF4076); anti-Laminin α1 was a gift from Dr. Dale Abrahamson (KU Medical Center, Kansas City, KS, rat monoclonal 8B3); anti-Laminin α2 and anti-β actin were from Sigma (St. Louis, MO, USA, Cat #: L0663); anti-Laminin α5 was a gift from Dr. Jeff Miner (Washington University, St. Louis, MO); anti-p-FAK^397^ was from Assay Biotechnology (Sunnyvale, CA, USA, Cat #: A0925) and from Invitrogen (Carlslab, CA); anti-Total FAK was from Cell Signaling Technology (Danvers, MA, USA, Cat #: 3285). Anti-MMP-10 antibodies were from Millipore (Billerica, MA, USA, Cat # ABT 289). All Alexa-fluor conjugated secondary antibodies were from Invitrogen (Carlsbad, CA), including donkey anti-rat 488, donkey anti-rabbit 555, goat anti-rat 488, goat anti-rabbit 555, donkey anti-rabbit 488, and donkey anti-goat 568. The small molecular inhibitor for FAK activation, TAE226 was from Chem Scene (Monmouth Junction, NJ, Cat #CS-0594); the peptide inhibitor for NF-kappaB (SN-50) was from Calbiochem (now EMD Millipore, Billerica, MA, Cat #481480)

### Immunofluorescence microscopy

Fresh frozen kidneys were sectioned at 8-µm and acetone fixed. Sections were incubated overnight at 4°C in primary antibody solution. The dual stain consisting of rat anti-mouse Laminin-α2 antibody (Sigma-Aldrich, St. Louis, MO) at 1∶200 and rabbit anti-mouse phospho-FAK^397^ antibody at 1∶25 as well as the dual stain of goat anti-mouse Integrin α8 antibody (R & D Systems, Minneapolis, MN) at 1∶1000 and rabbit anti-mouse Laminin-5 antibody, at 1∶1000 were diluted in 0.3% PBST+5% FBS. Rabbit anti-mouse Fibronectin antibody at 1∶300 and rat anti-mouse CD11b antibody at 1∶100 were diluted in 7% Milk. Slides were rinsed with 1X PBS and incubated with the appropriate Alexa Fluor donkey secondary antibodies at 1∶300 for 1 hour at room temperature. They were then rinsed again with 1X PBS and mounted with Vectashield Mounting Medium with DAPI (Vector, Burlingame, CA). The dual stain of mouse-anti rat Laminin α1 antibody at 1∶300 and rabbit anti-mouse phospho-FAK^397^ antibody at 1∶25 were diluted in 0.3% PBST+5% NGS and incubated overnight at 4°C. Slides were rinsed with 1X PBS and incubated with the appropriate Alexa Fluor goat secondary antibodies at 1∶300 for 1 hour at room temperature. They were then rinsed again with 1X PBS and mounted with Vectashield Mounting Medium with DAPI.

**Primary Mesangial Cells** were derived and characterized as previously described [9]. Three independent Transwell Migration Assays were performed using 0.5 µM TAE226 as previously described [11]. For pFAK^397^ Western Blot, cells were maintained on 1% FCS-containing media for two days, and then cultured overnight in 0.1% BSA (Fraction V, Roche Diagnonistics, Mannheim, Germany) and TAE226 added to 0.5 and 1.0 µM. After five hours protein was collected in M-PER (Thermo Scientific, Rockford, IL) containing Protease Inhibitor Cocktail P8340 at 1∶100 (Sigma, St. Louis, Mo), 5 mM Sodium Fluoride (Sigma), and 2 mM Sodium Orthovanadate (Sigma) and Western Blots run as described below.

**Conditionally Immortalized Glomerular Epithelial Cells (GEC's)**, were previously derived and characterized [4]. Cells were grown under permissive conditions (10% FCS, 10 U/ml γ-interferon at 33°C). Stable FAK and Scrambled Knock-Down GEC's were established as follows: 8.5 million cells were electroporated in 0.5 mls Gene Pulser Electroporation buffer (Bio-Rad Laboratories, Hercules, CA) containing 20 µg Silencer 4.1 CMV neo (Ambion, Austin, TX) plasmid expressing Ptk2 or scrambled siRNA, at .220 kV, 1.00 (µFx1000) in a 4 mm gap cuvette and incubated for 10 minutes on ice. Cells were plated under permissive conditions and 2 mg/ml G418 (Invitrogen, Carlsbad, CA) was added three days later. G418 selection was maintained for two weeks and clonal populations of selected cells generated by “limiting-dilution”. RNA and Protein was collected from expanded clonal populations placed under “non-permissive” conditions (5% FCS, no γ-interferon at 37°C) for two weeks, using Trizol (Invitrogen) and M-PER (Thermo Scientific) respectively. Plasmid(s) Expressing siRNA's were constructed using Ambion Silencer 4.1- CMV neo, Ambion Silencer Select siRNA Ptk2 (ID s65838) and Negative Control #1 (cat# AM4611) sequence(s) as per manufacturer's instructions.

### NF-ĸB Staining and -/+ Stretch pFAK^397^ Western Blot/Gene Expression

GEC's were differentiated under “non-permissive” conditions for ten days, plated onto Bioflex 6-well plates (Flexcell International, Hillsborough, NC) coated with Collagen Type 1 (rat tail, BD Biosciences, Bedford, Mass)/Placental Laminin (Sigma), cultured for two days in 0.5% FCS and exposed to mechanical strain for 4 hours, as previously described [15]. For NF-ĸB Staining, cells were fixed with 2% PFA, 4% Sucrose in PBS for 10 minutes, permeabilized with 0.3% Triton, as previously described [4], incubated with αNF-ĸB P65 antibody at 1∶50 overnight at 4°C,and then incubated with anti-rabbit secondary antibody at 1∶750 for two hours at room temperature. Gaskets were then cut out, mounted on slides with Vectashield (Vector Laboratories, Burlingame, CA) and cover slipped. For -/+ Stretch pFAK^397^ Western blots, protein was collected in M-PER (Thermo Scientific) as above and Western blot run as described below. For gene expression analysis, GECs were cultured on placental laminin and exposed to 20 hours of mechanical strain and RNA isolated as previously described [15].

**NF-ĸB SN50, Inhibitor Peptide Treatment**, GEC's were cultured as described above, 10 µM NF-ĸB SN50 Inhibitor Peptide (EMD Millipore, Billerica, MA) was added, and after one additional hour) exposed to 20 hours of mechanical strain and RNA collected as above.

### Basal Lamina and -/+ TAE226 pFAK^397^ Western Blots/Gene Expression

Following 10 days of differentiation, GEC's were cultured in 0.5% FCS for 2 days and plated onto tissue culture dishes previously coated with 50 µg/ml Collagen Type 1 (Rat tail, BD Biosciences) and 2 µg/cm^2^ Placental Laminin (Sigma) in 0.1% BSA containing media. For Basal Lamina experiment, additional dishes were coated with Collagen Type 1 and 1.25 µg/cm^2^ EHS Laminin (BD Biosciences) or 1.25 µg/cm^2^ Merosin (Chemicon, Temecula CA). For -/+ TAE266 experiment, 20 µM TAE226 was included in the media. Protein was collected 15 hours later in M-PER (Thermo Scientific) as above and Western Blot run as described below. For gene expression analysis, GECs were cultured on either placental laminin or merosin for 70 hours and RNA was isolated and analyzed as described above.

### Confocal microscopy

Slides were coverslipped using Vectashield mounting medium containing DAPI to counter-stain the nuclei (Vector, Burlingame, CA) and confocal images captured using a Zeiss AxioPlan 2IF MOT microscope interfaced with a LSM510 META confocal imaging system, using a 63X NA:1.4 oil objective. Final figures were assembled using Adobe Photoshop and Illustrator software (Adobe Systems, CA).

### Glomerular isolation

Glomeruli were isolated by perfusing animals with Dynal M-450 beads (Life Technologies, Hercules, California) and isolating the glomeruli using a magnet as described previously [4].

### Real time qRT-PCR

Total RNA was reverse transcribed using SuperScript III (Invitrogen, Life Technologies, Grand Island, NY) with Oligo(dT)_20_ Primer (Invitrogen). The real time PCR was carried out using TaqMan Gene Expression Master Mix (Applied Biosystems, Life Technologies, Grand Island, NY), and quantified using StepOnePlus Real-Time PCR System (Applied Biosystems). Samples were normalized to Mouse GAPDH Endogenous Control VIC Probe (Applied Biosystems catalogue #4352339E) which was run alongside MMP-9 (Catalog #4331182, ID# Mm00442991_m1), MMP-10 (Catalog #4331182, ID# Mm00444630_m1), MMP-12 (Catalog #4331182, ID# Mm00500554_m1), IL-6 (Catalog #4331182, ID# Mm00446190_m1), NFKbia (Catalog #4331182, ID# Mm00477798_m1), and FAK (Catalog #4331182, ID# Mm00433209_m1) TaqMan Gene Expression Assay Probes (Applied Biosystems). Each of the samples were run in triplicate with a final reaction volume of 20 µl with the following cycling parameters: 50°C for 2 min, 95°C for 10 min, followed by 40 cycles of a two-step PCR consisting of 95°C for 15 s and 60°C for 1 min. Relative changes in gene expression were determined by calculating the fold change using the comparative C_T_ method of 2^-ΔΔCT^. Data are expressed as the mean with standard deviation for at least four independent RNA samples per data point.

### Immunoblotting

Ten to fifteen micrograms, of cellular protein, was resolved in a 10% SDS-PAGE and then electrotransfered to PVDF membrane. The membranes were cut in half and the upper half (250 kDa to 75 kDa) used for pFAK/tFAK immunoblotting while the bottom half used for β-actin immunoblotting (loading control). Conditions for pFAK detection: the membrane was blocked in milk blocking solution (5% milk containing 0.2% Tween-20 in PBS) for 1 hour at room temperature with constant shaking and incubated overnight at 4°C with anti-pFAK 1∶1,000 in BSA blocking solution (1% BSA containing 0.2% Tween-20 in PBS). After several washes the membrane was incubated with a goat anti-rabbit HRP conjugated secondary antibody (1∶20,000) in BSA blocking solution for 1 hour at room temperature. Conditions for tFAK detection: the same membrane used for pFAK immunoblot was stripped and re-probed for tFAK. The blocking was done in 5% milk blocking solution for 1 hour at room temperature, followed by an overnight incubation with the tFAK primary antibody (1∶500) in milk blocking solution. After several washes the membrane was incubated with a goat anti-rabbit HRP conjugated secondary antibody (1∶3000) in 5% milk for 1 hour at room temperature. Conditions for β-actin detection: the membrane was blocked for 1 hour with 10% milk blocking solution and then incubated overnight with the mouse anti-β-actin (1∶2,000) in the same blocking solution. After several washes the membrane was incubate with a goat anti-mouse HRP-conjugated secondary antibody (1∶3,000) in 10% milk for 1 hour at room temperature. After several washes the membrane was developed using Pierce ECL Western Blotting Substrate (Thermo Scientific, Rockford, IL) as per manufacturer's direction.

### Treatment of mice with TAE226

Four Col 4A3 knockout mice from 129 Sv/J background were given 50 mg/Kg TAE226 (ChemScene, LLC Monmouth Junction, NJ) 1 X daily by oral gavage starting at two weeks of age until seven weeks old. The TAE226 was diluted in a 0.5% carboxymethylcellulose suspension. Three control Col 4a3 knockout mice of the same age were given 0.5% CMC suspension (vehicle) alone and served as controls.

### Albumin and creatinine assays

Urine was collected weekly and albumin concentrations were analyzed as instructed using a mouse albumin ELISA kit #MSAKT from Molecular Innovations (Novi, MI). Albumin levels were normalized to creatinine using QuantiChrom Creatinine Assay Kit (DICT-500) (BioAssay Systems, Hayward, CA) according to the manufacturer's instructions.

### Transmission electron microscopy

Transmission electron microscopy was performed as described previously [8].

### Statistical analysis

Real time qRT-PCR and western blot data were analyzed using the one sample Students t-test (controls were always set arbitrarily equal to 1). Proteinuria and BUN data were analyzed by two tailed Students t-test.

## Results

**Figure 1:**
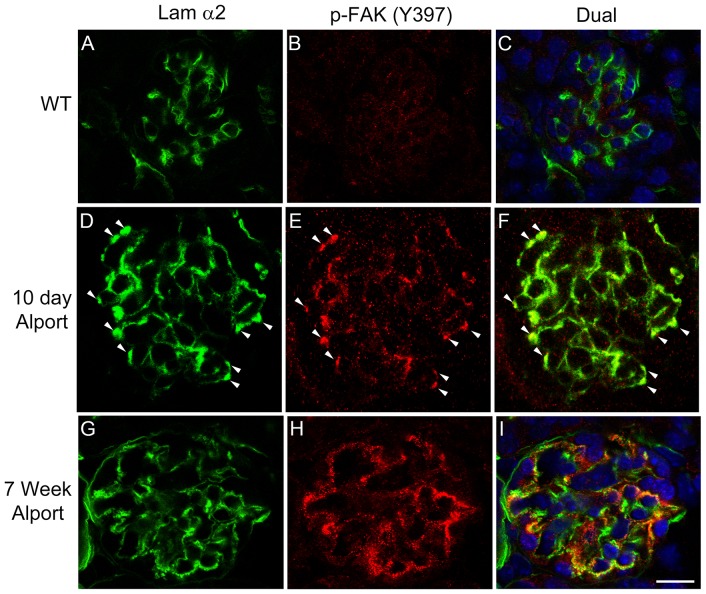
Activation of focal adhesion kinase occurs specifically in regions of the GBM where laminin α2 is present, and is a very early event in Alport glomerular pathogenesis. Cryosections from 10 day old Alport mice (D–F), 7 week old Alport mice (G–I), and wild type littermates (A–C) were immunostained with antibodies specific for the α2 chain of laminin or pFAK^397^. Arrowheads denote areas of dual immunostaining in the glomerular capillary loops. Scale bar = 15 µm.

In earlier work we and others showed that laminin α2 accumulates in the GBM of Alport mice, dogs and humans [8], [10]. In **Figure 1**, we show that the appearance of laminin α2 in the GBM correlates with the activation of FAK in glomerular podocytes. **Figure 1** panels A-C show that in wild type mice laminin α2 is restricted to the mesangium and no appreciable level of FAK activation (as determined by immunostaining for pFAK^397^) is observed. As early as 10 days of age in 129 Sv/J autosomal Alport mice we begin to observe punctate immunostaining for laminin α2 in the GBM of some (30-50%) glomeruli (**Figure 1** panel D, arrowheads). Wherever we observe GBM laminin staining we also see immunopositivity for pFAK^397^ (**Figure 1** panels E and F), indicating activation of FAK specifically in regions of the GBM where laminin α2 has been deposited. By 7 weeks of age in Alport glomeruli, GBM laminin α2 is more extensively observed in the GBM (**Figure 1** panel G), and continues to localize adjacent to podocyte pFAK^397^ immunostaining (**Figure 1** panels G-I). Dual immunostaining of glomeruli for pFAK^397^ and the podocyte marker WT1 confirms that laminin α2-mediated FAK activation is occurring specifically in podocytes (Figure S1).

**Figure 2:**
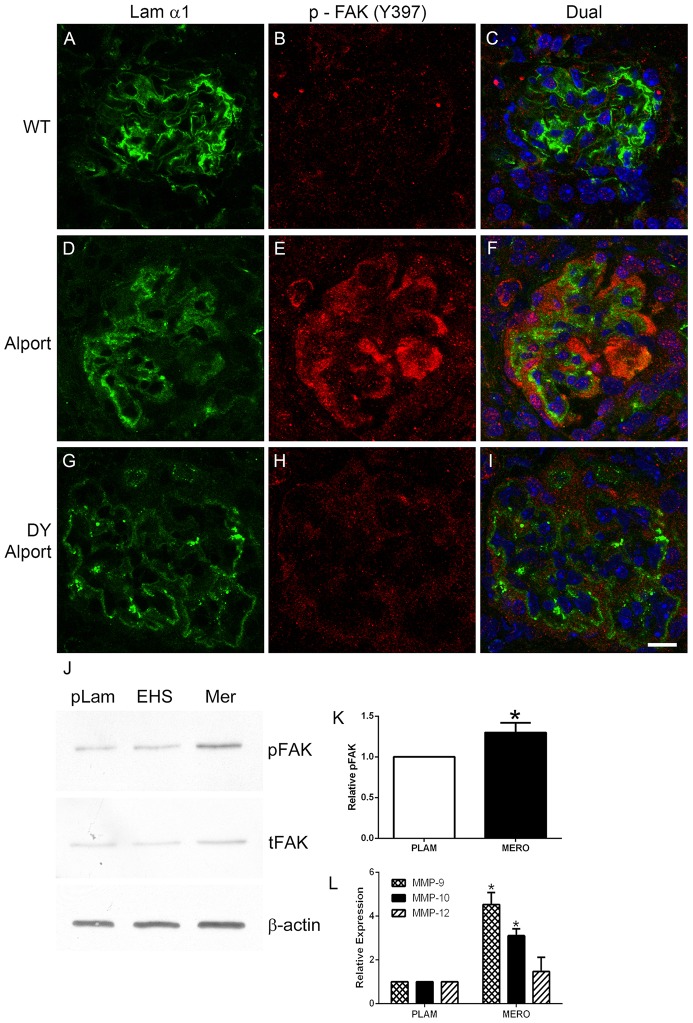
Laminin α2, but not laminin α1 activates FAK on podocytes *in vivo* and *in vitro*. Panels A–C; 7 week old wild type glomerulus stained with antibodies specific for laminin 111 and pFAK^397^ show absence of pFAK immunostaining. Panels D–F; 7 week Alport glomerulus stained with antibodies specific for laminin α1 and pFAK^397^ pFAK immunostaining in podocytes adjacent to laminin α1-immunopositive GBM. Panels G-I show the same immunostaining as for D–F using Alport mice that do not express laminin α2 (the dy/dy muscular dystrophy mutation). Note the absence of pFAK^397^ immunostaining even though GBM is immunopositive for laminin α1. Panel J. Wild type podocytes were differentiated for 2 weeks and then plated on placental laminin, EHS laminin, or merosin for 15 hours. Extracts were prepared and analyzed by western blot for expression of pFAK^397^ and total FAK. β-actin was used as a loading control). Panel K shows quantitative analysis of pFAK397 relative to total FAK for several western blots. Panel L shows real time qRT-PCR results for transcripts endocing the indicated MMPs, demonstrating significantly elevated expression of MMP-9 and MMP-10 for cells cultured on merosin (MERO) relative to cells cultured on placental laminin (PLAM). Scale bar = 10 µm.

In addition to laminin α2, laminin α1 has also been shown to accumulate in the GBM of Alport mice [16]. To determine whether laminin α2 and/or laminin α1 is responsible for activation of FAK in glomerular podocytes we crossed the 129 Sv/J autosomal Alport mouse with a laminin α2-deficient mouse (a model for muscular dystrophy), also on the 129 Sv/J background. As evidenced in **Figure 2**, while wild type mice show no FAK activation (A-C), the 7 week old Alport mouse shows FAK activation in podocytes bound to laminin α1-immunopositive GBM (**Figure 2** D-F), the age matched laminin α2-deficient Alport mouse (DY Alport), while immune-positive for laminin α1 (**Figure 2** panel G), does not show appreciable FAK activation anywhere in the glomerulus (**Figure 2** panel H). To assess in a more direct manner whether laminin α2 activates FAK in podocytes, we cultured differentiated conditionally immortalized podocytes on placental laminin (primarily laminin 521), EHS laminin (laminin 111), and merosin (laminin 211) for 15 hours and analyzed cell lysates for total FAK and pFAK^397^. The results in **Figure 2** panel J and K show significantly higher levels of pFAK^397^ in podocytes cultured on merosin compared to either placental laminin or EHS laminin, indicating that laminin 211 can activate FAK directly in these cells.

**Figure 3:**
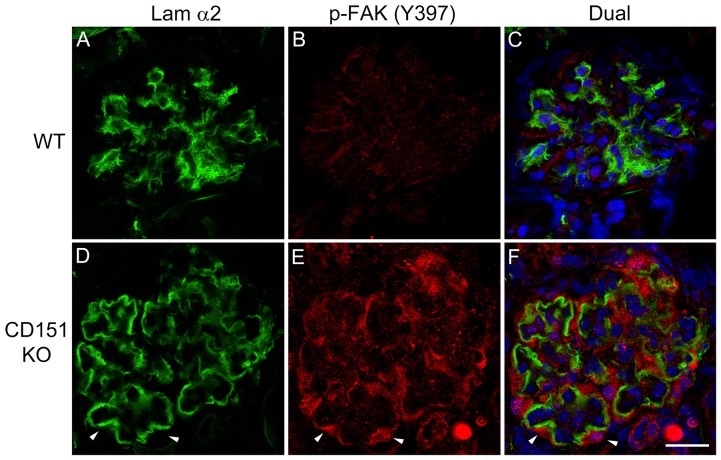
Activation of focal adhesion kinase occurs specifically in regions of the GBM where laminin α2 is present in CD151 knockout mice. Cryosections from 10 week old CD151 knockout mice (D–F) and wild type littermates (A–C) were immunostained with antibodies specific for the α2 chain of laminin or pFAK^397^. Arrowheads denote areas of dual immunostaining along the capillary loops. Scale bar = 15 µm.

In a recent paper we showed that laminin α2 also accumulates in the GBM of CD151 knockout mice [11]. If laminin α2 is responsible for FAK activation on glomerular podocytes *in vivo* we would expect to observe pFAK^397^ immunostaining at the interface of podocyte binding to the GBM in these mice as well. **Figure 3** shows that this is indeed the case. **Figure 3** D-F clearly demonstrate laminin α2 immunostaining in the GBM with clear presence of pFAK^397^ in podocytes adjacent to laminin α2-immunopositive GBM, consistent with laminin α2 mediated FAK activation in the podocytes of CD151 knockout mice. This data further supports that FAK activation results from the presence of laminin α2 in the GBM, since unlike Alport mice which lack GBM collagen α3(IV), α4(IV) and α5(IV) chains, CD151 knockout mice have otherwise normal GBM composition.

**Figure 4:**
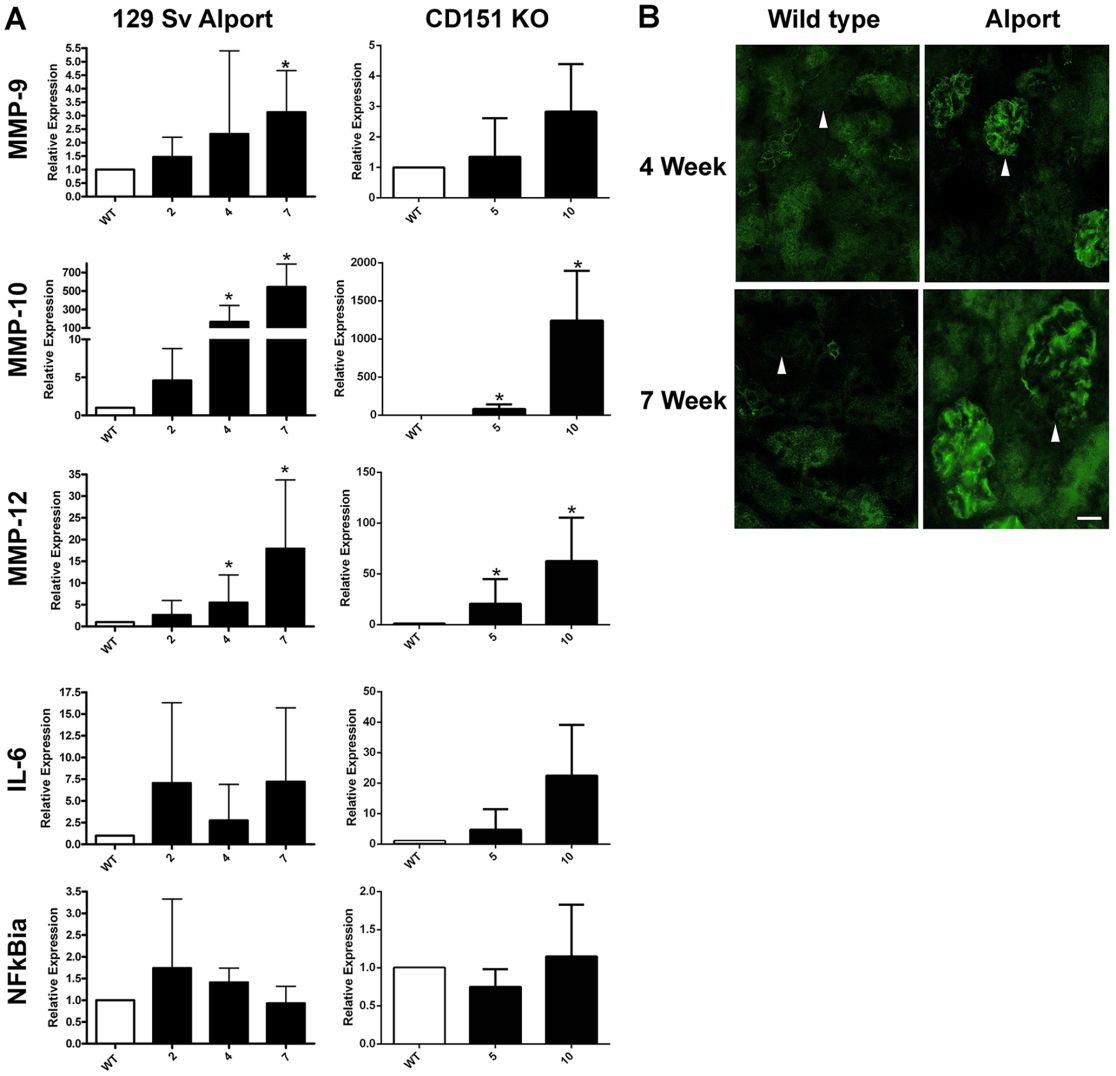
Induction kinetics for MMP-9, MMP-10, MMP-12, IL-6, and NF-kappaBia in glomeruli from Alport mice and CD151 knockout mice. Panel A. Glomeruli were isolated from CD151 knockout mice and Alport mice along with strain/age matched wild type littermates at the indicated ages using bead isolation. Total glomerular RNA was analyzed by real time RT-PCR using primers specific for the indicated transcripts. Each data point represents at least five independent samples. Significant differences when comparing the data from mutants with wild type littermates are denoted with asterisks (p<0.05). Note that IL-6 and NF-kappaBia did not reach significance likely due to a large variance in the data, but trended towards significance. Panel B shows that MMP-10 protein is induced in Alport glomeruli at both 4 and 7 weeks of age as determined by immunofluorescence analysis. Scale bar = 15 µm.

Previous studies from our lab and others have demonstrated a clear link between the induction of matrix metalloproteinases and glomerular basement membrane damage in Alport mice [4], [5], [9]. Based on Affymetrix analysis of wild type and Alport glomerular RNA from 129 Sv/J mice, we determined that MMP-9, MMP-10, and MMP-12 were significantly induced in the Alport glomeruli. MMP-10 and 12 are massively induced (700- and 40-fold, respectively), suggesting that these MMPs might be principally responsible for the GBM damage observed in Alport mice. Given that previous studies in other systems have linked FAK activation to the induction of MMPs [12], [17], we surmised that we should observe parallel dysregulation in glomerular RNA from Alport mice and CD151 knockout mice, if indeed podocyte MMP induction is linked to FAK activation. We profiled glomerular mRNA expression for a timecourse in both models using real time qRT-PCR. The results in **Figure 4** panel A demonstrate significant and progressive induction of all three MMPs in both models. The strikingly robust induction of MMP-10 and MMP-12 observed in Alport glomeruli is also observed in the CD151 knockout mouse, suggesting that these transcripts are induced via the laminin α2-mediated FAK activation pathway. Since earlier work demonstrates FAK-mediated induction of MMPs via activation of NF-kappaB [18], [19], we also examined NF-kappaBia transcript, which serves as an indicator for the state of NF-kappaB activation [20]. As shown in **Figure 4**, neither NF-kappaB or the NFkappaB-responsive pro-inflammatory cytokine IL-6 [21] transcripts show significant induction due to a high degree of variability in abundance, likely owing to multiple pathways (in addition to FAK) converging on the activation of NF-kappaB. Further supporting a direct role for laminin α2-mediated FAK activation in the induction of these MMPs in podocytes, **Figure 2** Panel L shows significantly elevated expression of MMP-9 and MMP-10 mRNAs from podocytes cultured on merosin relative to podocytes cultured on placental laminin.

MMP-10 expression in the glomerulus has not been previously documented. To further qualify the validity of the qPCR results, we analyzed cryosections of 4 and 7 week old wild type and Alport mice for MMP-10 expression by immunofluorescence. The results in **Figure 4** panel B show that MMP-10 is not detected in wild type glomeruli, but is robustly expressed in Alport glomeruli at both early and advanced disease states. This staining is not due to cross reactivity with MMP-3 because MMP-3 is not induced in Alport glomeruli (data not shown).

**Figure 5:**
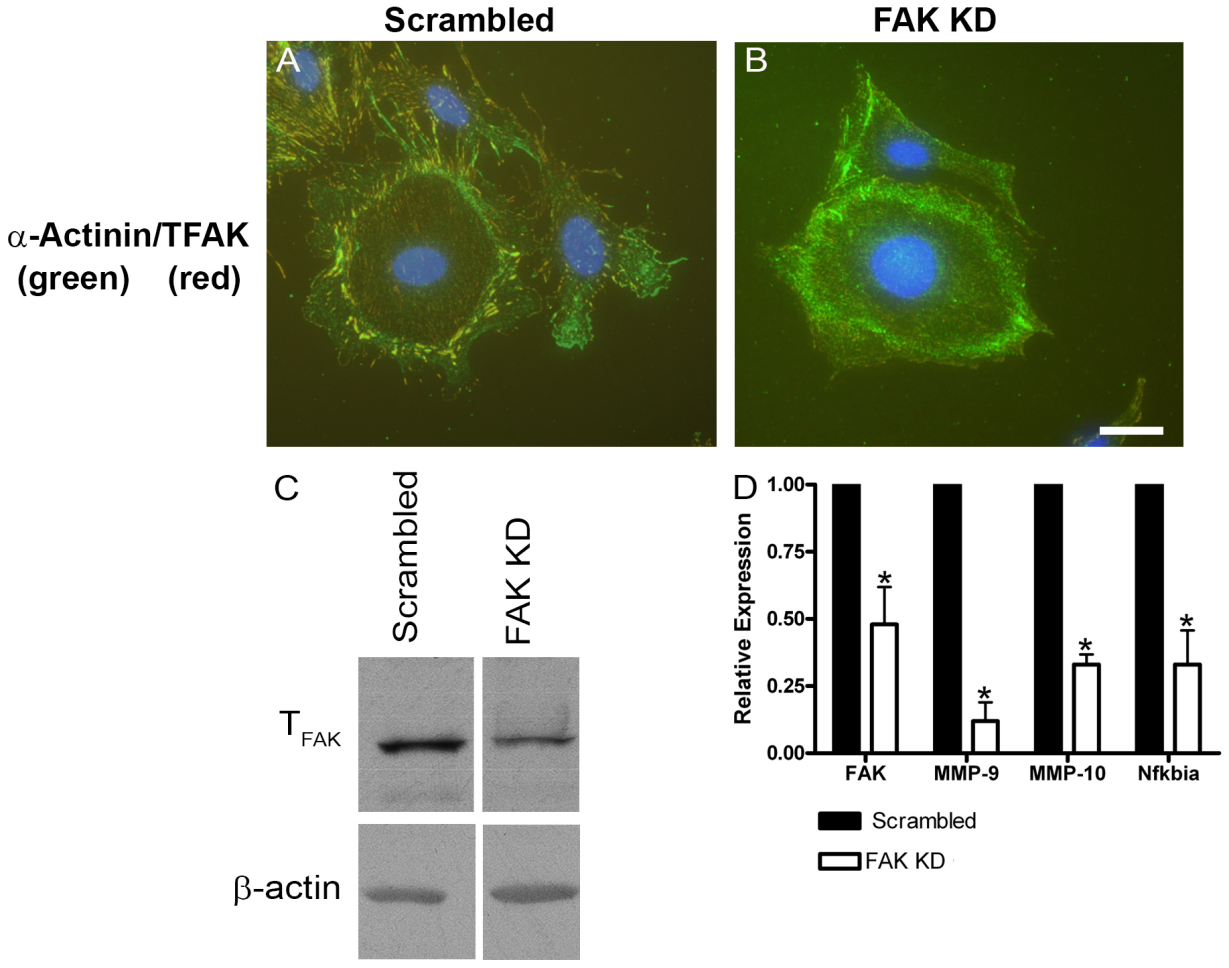
Stable siRNA knock-down of FAK in cultured podocytes results in significantly reduced expression of MMP-9, MMP-10, and NF-kappaBia. Conditionally immortalized podocyte cell cultures were transfected with vector encoding a siRNA expression cassette for FAK. A vector encoding a scrambled siRNA was used as a control. Stable clones were selected and propagated. The data presented is representative of several independently selected clones. Panels A and B show that while cells expressing the scrambled vector still have robust focal adhesions (panel A), they are significantly reduced or absent in the cells expressing the FAK siRNA (panel B). Western blot for total FAK confirms a reduction of FAK protein in the FAK siRNA transfected cultures (panel C). Real time qRT-PCR analysis of RNA from these clones shows a significant reduction in the expression of mRNAs encoding FAK, MMP-9, MMP-10, and NF-kappaBia in FAK siRNA expressing cells versus those expressing the scrambled siRNA. Scale bar = 15 µm.

To more directly establish the link between FAK activation and MMP gene expression in glomerular podocytes we performed siRNA knockdown of FAK in conditionally immortalized podocyte cell cultures. Stable clonal populations of siRNA knockdown podocyte cell lines were established. The best knockdown observed in these stable clones was about 60%. **Figure 5** shows results typical for several clones examined. In **Figure 5** panel B, note the relative absence of focal adhesions in podocytes cultured on rat tail collagen relative to the parent podocyte cell line shown in Panel A. **Figure 5** panel C shows that total FAK protein is reduced in extracts from the siRNA knockdown cells relative to cells transfected with a scrambled siRNA construct. **Figure 5** panel D shows that FAK knockdown cells show significantly reduced expression of MMP-9, MMP-10, and NF-kappaBia, further supporting the link between FAK activation and induction of these MMPs in glomerular podocytes. Interestingly MMP-12 was not significantly reduced in the knockdown cells (data not shown).

**Figure 6:**
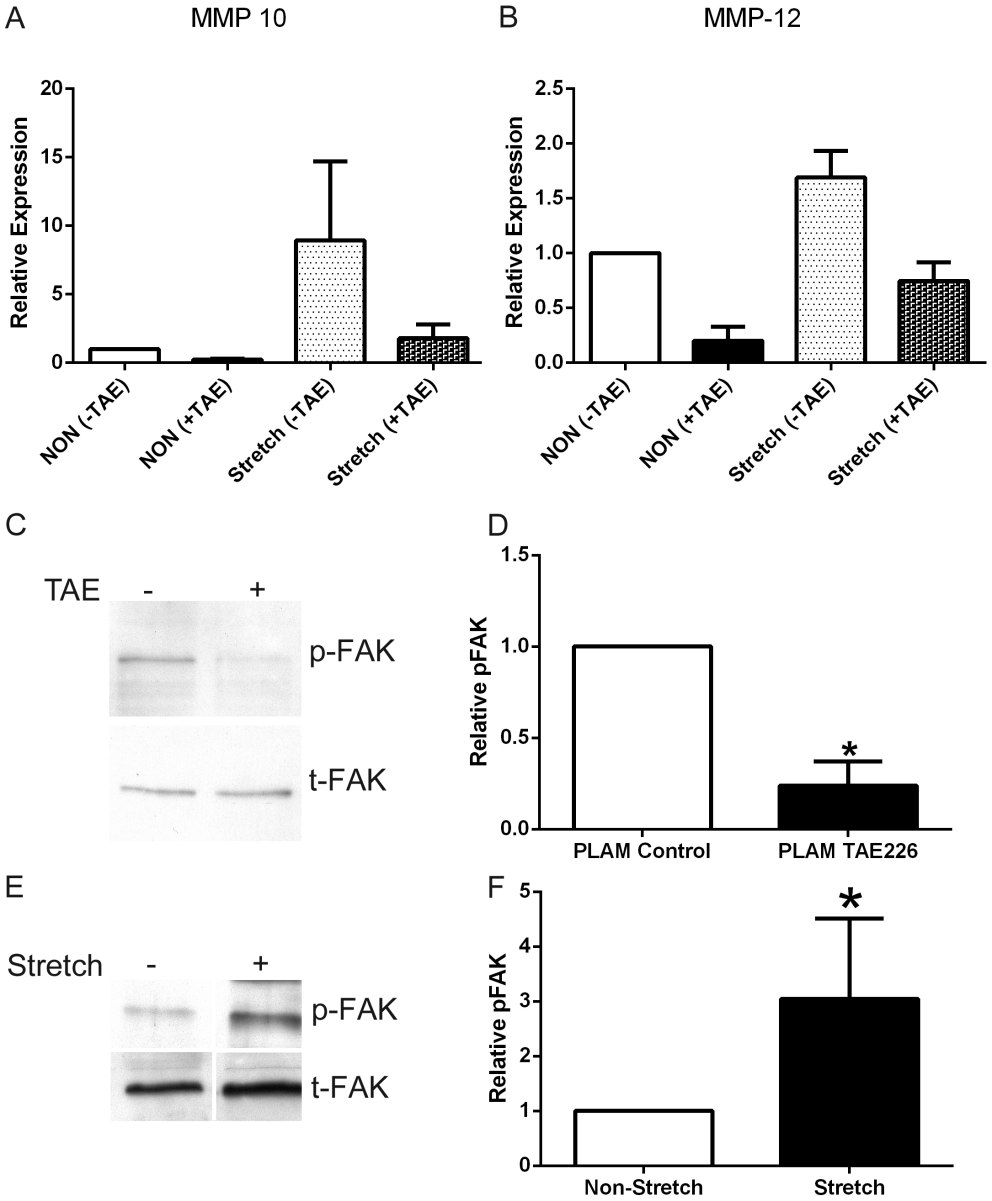
The small molecule inhibitor for FAK, TAE226, reduces FAK activation and stretch-induced MMP-10 and MMP-12 expression in cultured podocytes. Panels A and B, cells were treated or not with TAE226 under static and stretched conditions and mRNA analyzed by real time qRT-PCR for the indicated transcripts. Panel C, Podocytes were cultured on placental laminin overnight and TAE226 added 1 hour before stretching. Extracts were prepared and analyzed by western blot for expression of pFAK^397^ and total FAK. Panel E, FAK activation was also analyzed by western blot of podocyte extracts from stretched and non-stretched cells, demonstrating that biomechanical stretching directly activates FAK. Panels D and F, Several replicate blots were scanned and quantified for pFAK relative to total FAK and statistically analyzed. Statistically significant differences are indicated with asterisks where p<0.05.

An alternative means of reducing FAK activation is by way of small molecule inhibitors. One such inhibitor, TAE226, has previously been shown to protect against glomerular injury by either lipopolysaccharide or anti-GBM antibody administration [22]. We cultured podocytes in the presence or absence of TAE226 to assess the effect on MMP expression. Since we have previously documented a role for biomechanical strain in the induction of MMPs and the acceleration of glomerular disease in Alport mice [11], [15] we assessed the effect of FAK inhibition by TAE226 on biomechanical stretch-mediated induction of MMP-10 and MMP-12. **Figure 6** panels A and B show, consistent with our earlier findings, that biomechanical stretch induced both MMP-10 and MMP-12, and that message levels for these two MMPs are reduced in cells stretched in the presence of TAE226 relative to untreated cells. **Figure 6** panel C and E shows that cell stretching induces the level of FAK activation and that treatment of cultured podocytes with TAE226 reduced the activation state of FAK as determined by western blot for pFAK^397^ protein. Analysis of multiple blots followed by densitometry (panels D and F) show that these effects on the activation state of FAK are significant.

**Figure 7:**
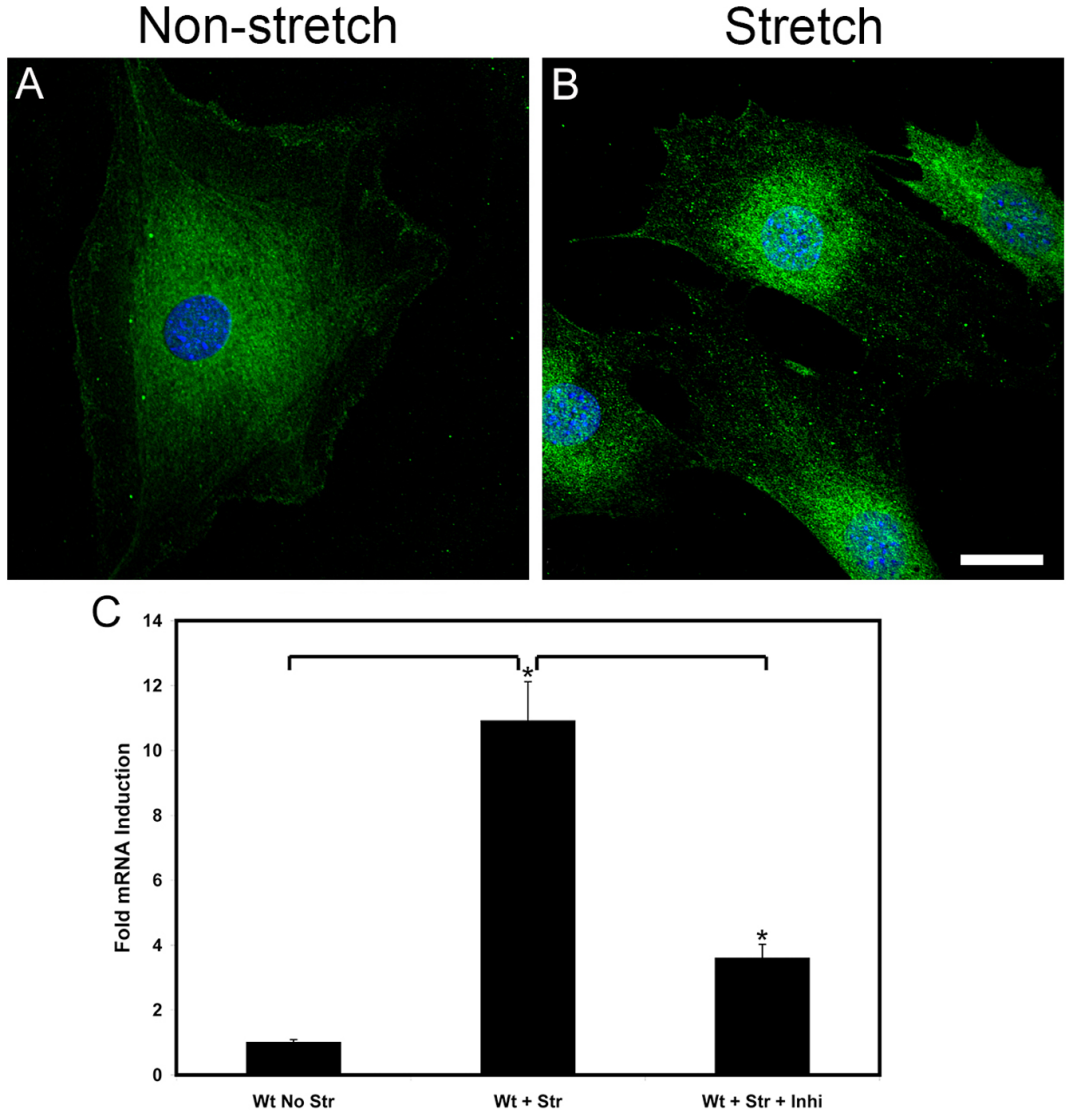
Biomechanical stretching activates NF-kappaB which regulates MMP-10 expression in cultured podocytes. NF-kappaB localizes primarily to the cytosol in non-stretched cultured podocytes (panel A). Subjecting the cells to cyclic biomechanical stretching results in the nuclear localization of NF-kappaB (panel B), which is consistent with its activation. Panel C shows that stretch-mediated induction of MMP-10 is blocked by addition of a peptide inhibitor for NF-kappaB to the culture medium. Scale bar = 20 µm.

In **Figure 7** panel B we show that biomechanical stretching results in the nuclear localization of NF-kappaB, which is consistent with NF-kappaB activation. Nuclear localization of NF-kappaB is not observed in non-stretched cells (panel A). Stretch-mediated induction of MMP-10 is attenuated by treating cells with a peptide inhibitor for NF-kappaB during the cyclic mechanical stretching (**Figure 7** panel C), demonstrating that MMP-10 induction may indeed be mediated by NF-kappaB activation.

**Figure 8:**
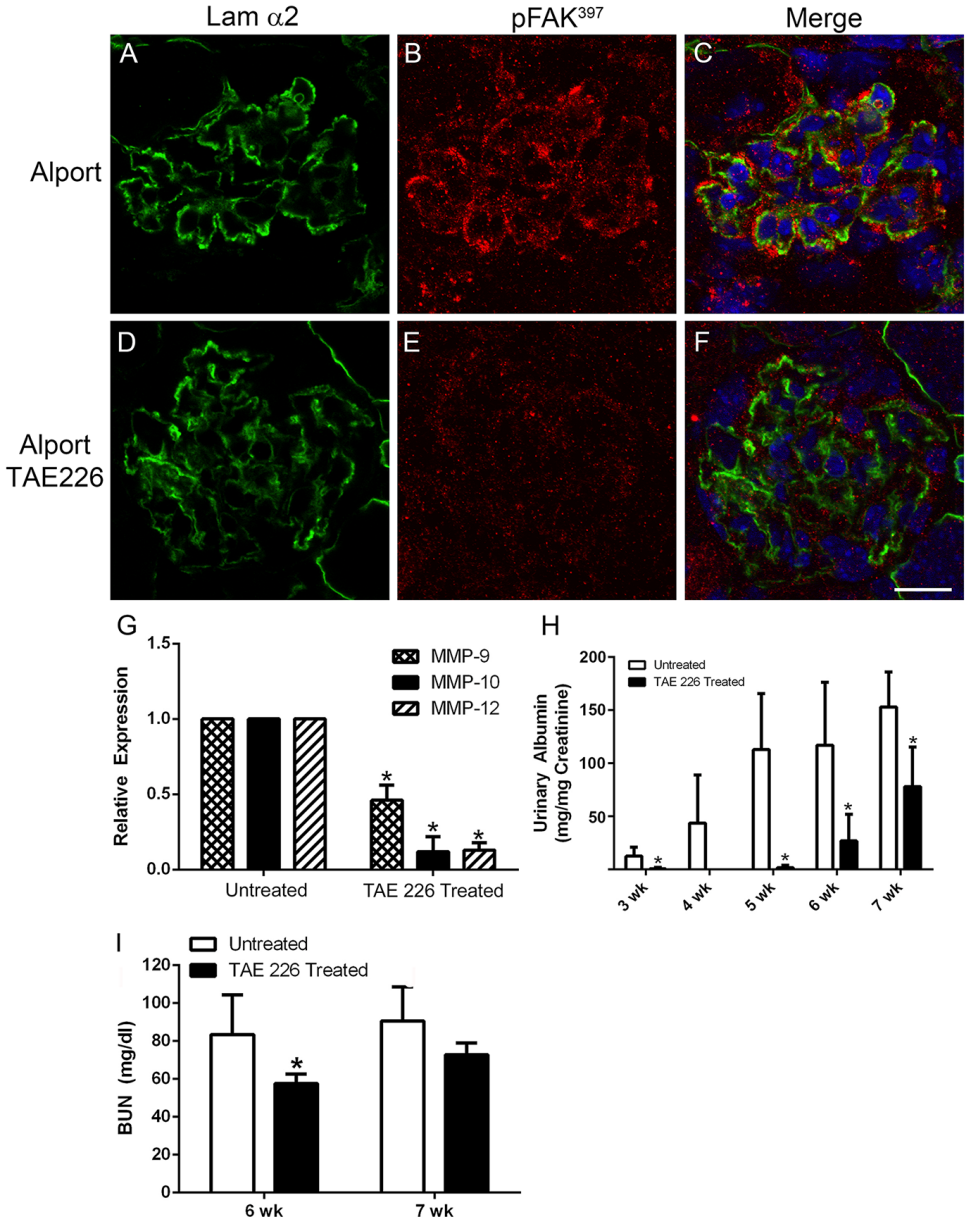
Treatment of Alport mice with the small molecule inhibitor for FAK, TAE226, blocks FAK activation, significantly reduces glomerular expression of MMP-9, -10, and -12, and ameliorates proteinuria and blood urea nitrogen levels. 129 Sv/J autosomal Alport mice were treated with TAE226 from 2 to 7 weeks of age. Panels A–F show that while pFAK^397^ immunostaining is present in podocytes adjacent to laminin α2-immunopositive basement membranes in vehicle treated mice, it is absent in mice treated with TAE226, indicating effective blockade. Real time qRT-PCR analysis of glomerular RNA in panel G shows significant reduction in expression of MMP-9, MMP-10, and MMP-12 in TAE226 treated mice relative to those given vehicle. Panel H and I show significant amelioration of proteinuria and BUN in treated mice, indicative of improved glomerular function. Scale bar = 15 µm.

To determine the role of laminin α2-mediated FAK activation on the progression of glomerular disease we treated autosomal Alport mice with TAE226 from 2 weeks of age (before the onset of proteinuria) to 7 weeks of age (near end stage). One kidney was used for glomerular RNA isolation by perfusion with Dynal M-450 beads, and the other prepared for histological and transmission electron microscopic (TEM) analysis. **Figure 8** panels A-C show FAK activation in podocytes adjacent to laminin α2 in the Alport GBM (denoted with arrowheads). Treatment with TAE226 abolished pFAK immunostaining (panels D-F) demonstrating effective *in vivo* blockade of FAK activation achieved through drug treatment. **Figure 8** panel G shows that FAK inhibition significantly reduced the mRNA expression levels for MMP-9, MMP-10, and MMP-12 relative Alport mice given vehicle. Panel H and I shows a significant reduction in proteinuria and blood urea nitrogen levels in treated Alport mice relative to Alport mice given vehicle. Lifespan studies were not conducted because TAE226 treatment resulted in significant growth stunting indicating a toxic side effect.

**Figure 9:**
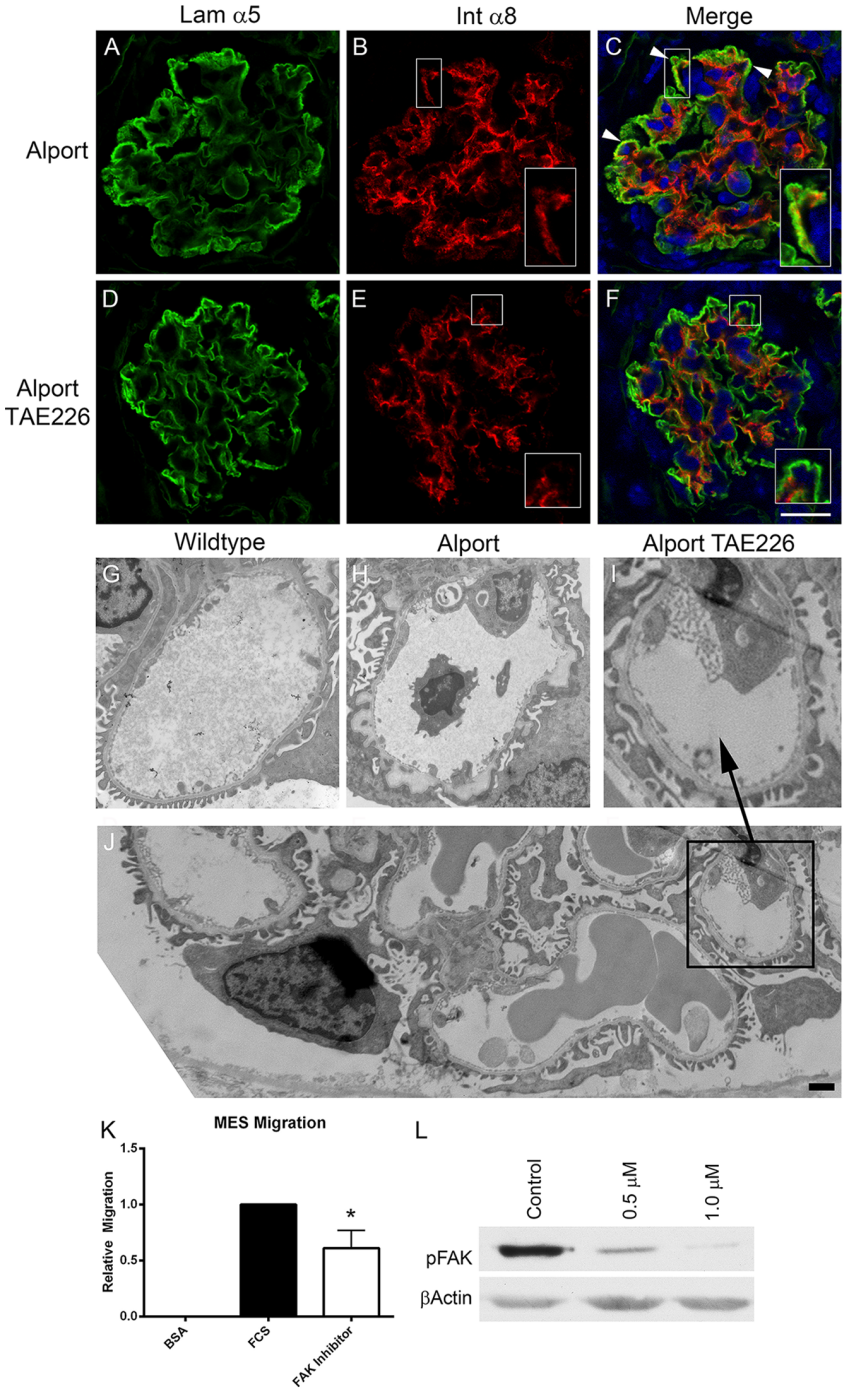
Treatment of Alport mice with TAE226 reduces mesangial process invasion of the glomerular capillary loops, ameliorates GBM ultrastructural dysmorphology, and significantly reduces pFAK activation and migratory potential of primary cultured mesangial cells. The same mice as in **Figure 8** were dual immunostained with the GBM marker, laminin α5, and the mesangial cell marker, integrin α8. Arrowheads in panel C denote regions where invasion of the capillary loops by mesangial processes is evident (panels B and C, inserts). This characteristic is markedly reduced in the TAE226-treated glomeruli where integrin α8 immunostaining is restricted to the mesangial angles (panels E and F. inserts). Transmission electron microscopic analysis shows that TAE226 treatment (Panel I) reduces the ultrastructural damage to the GBM normally present by 7 weeks of age in this model (panel H). Panel J is provided to indicate that amelioration of GBM dysmorphology is generally observed. Panel K shows that treatment of primary cultured mesangial cells with TAE226 significantly reduces their migratory potential relative to untreated cells. Panel L shows a dose response for FAK inhibition by TAE226 in cultured mesangial cells. Panel F, scale bar = 15 µm; Panel J, scale bar = 2 µm.

In a recent and related publication we showed progressive mesangial invasion of the glomerular capillary loops in Alport mice [11]. We provide ultrastructural evidence which further supports this novel observation where we demonstrate immunogold labeling for integrin α8 (robustly expressed on mesangial cells) in blebs that are frequently observed in the subendothelial region of the glomerular capillaries of Alport mice, but not wild type mice (Figure S2). **Figure 9** panels A-F show dual immunofluorescence staining for the GBM marker laminin α5 and the mesangial cell surface marker integrin α8. Mesangial processes in the capillary loops are clearly observed in the vehicle treated Alport glomeruli (**Figure 9** panel C, arrowheads, inset panel). TAE226 treatment resulted in amelioration of mesangial process invasion (panels E and F, inset panels; showing integrin α8 immunostaining only at the mesangial angles), suggesting that FAK activation on mesangial cells, likely due to biomechanical stretching, may contribute to this process mechanistically. Consistent with this notion, treatment of mesangial cells with TAE226 significantly reduced their cell migratory potential (**Figure 9** **panel K**) and blocked pFAK activation in a dose-dependent manner (**Figure 9** **panel L**). Transmission electron microscopic analysis of the GBM in TAE226-treated animals showed markedly improved GBM architecture relative to mice given vehicle (**Figure 9** **panels G-J**).

**Figure 10:**
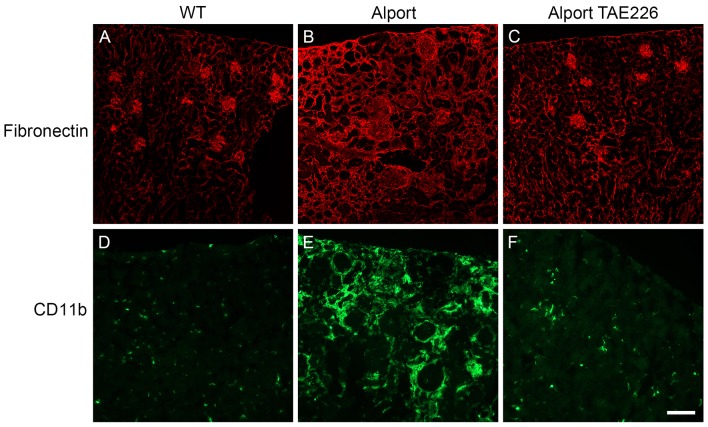
Treatment of Alport mice with TAE226 ameliorates interstitial fibrosis and monocyte infiltration. Kidney cryosections from wild type and Alport mice that were either treated with vehicle or TAE226 were immunostained with antibodies specific for fibronectin (A–C or the monocyte marker, CD11b (D–F). The accumulation of fibronectin in the interstitium, indicative of fibrosis, while abundant in Alport mice (panel B) is not apparent in Alport mice treated with TAE226 (panel C), which appear similar to wild type mice (panel A). Similarly, monocyte infiltration, as indicated by CD11b immunopositive cells, is readily apparent in Alport mice (panel E). In TAE226-treated Alport mice (panel F), however, the abundance of monocytes is similar to that in wild type mice (panel D), which are resident cells rather than infiltrating cells. Scale bar = 50 µm.

To evaluate the effect of TAE226 treatment on renal fibrosis we stained kidney sections with antibodies specific for either fibronectin (to assess renal scarring) or CD11b (to assess for monocytic infiltration). The results in **Figure 10** show that TAE226 treatment results in remarkably robust reduction in both renal scarring (compare **Figure 10** A–C) and monocytic infiltration (**Figure 10** panel D–F). In all four treated mice it was difficult to distinguish immunostaining of wild type kidneys from TAE226 treated Alport kidneys using these two antibodies.

## Discussion

The initially punctate and then progressive deposition of laminin α2 and laminin α1 in the GBM of Alport mice is a phenomenon that would be expected to have some consequence contributing to the progressive deterioration of the glomerular structure/function, although until now no definitive functional consequence has been described. Here we provide evidence that laminin α2 activates FAK on glomerular podocytes resulting in downstream activation of MMPs and pro-inflammatory cytokines that contribute to the progressive glomerular pathogenesis. At least some of these genes appear to be induced by NF-kappaB activation, suggesting that the laminin α2/FAK/NF-kappaB circuit might be a central player driving the progression of Alport glomerular disease. In support of this notion, treatment of Alport mice with a small molecule inhibitor for FAK, TAE226, resulted in a significant reduction in glomerular expression of MMP-9, MMP-10, and MMP-12, improved glomerular function, ameliorated ultrastructural damage to the GBM, and blocked interstitial monocyte infiltration and interstitial fibrosis.

While the effects of TAE226 on FAK activation in glomerular podocytes is likely the principal contributing factor underlying the observed improvement of the GBM ultrastructure and function, it is also likely that the systemic administration of this compound might have multiple influences on improved renal health in these animals. For example, we recently showed that laminin α2 is deposited in the GBM by mesangial processes that invade the glomerular capillaries [11]. In the current study, we show that TAE226 treatment reduced the degree of mesangial process invasion (**Figure 9** panels D–F) and reduces the migratory potential of cultured mesangial cells (**Figure 9** panel J), suggesting that mesangial processes invasion of glomerular capillaries in Alport syndrome might be partially FAK-dependent. This makes some sense considering that deletion of integrin α1β1, which is present on mesangial cells [23], also ameliorates mesangial process invasion of the glomerular capillaries in Alport mice, and significantly improves renal health in this model [8], [11]. The remarkable reduction in interstitial monocytes might reflect a third distinct activity for FAK in Alport renal disease. In earlier work we showed that interstitial monocytes in our mouse model are primarily α1β1 integrin-positive [24]. We later showed that α1β1 integrin-positive monocytes are selectively trafficked to the interstitium in Alport kidneys [25]. In other systems it has been shown that leukocyte activation following tight binding to the vascular endothelium can be mediated through FAK signaling [26]. Thus inhibiting monocyte activation to reduce interstitial monocyte efflux might represent a third target of FAK inhibitors that improves renal health in Alport mice.

The current study demonstrates using both *in vitro* (**Figure 2G**) and *in vivo* (**Figure 2A-F**) approaches that laminin α2, but not laminin α1, activates FAK on glomerular podocytes. This is an important distinction when one considers that abnormal laminins have been shown to accumulate in the GBM in patients with membranous glomerulonephritis [27], [28] where much like the Alport model, the laminins are first observed in the irregularly thickened regions of the GBM. In the Alport mouse model, irregularly thickened abnormal laminin-rich regions of the GBM were shown to be more permeable to injected ferritin, suggesting that these regions are comprised of loosely assembled matrix that might contribute to progressive leakiness and proteinuria [2]. Based on our observation of FAK mediated induction of MMP-9, MMP-10, and MMP-12 in the Alport podocytes, we would argue that the increased GBM permeability in these thickened regions might reflect partially degraded GBM. Earlier studies found that deletion of MMP-9 in Alport mice did not influence renal disease progression, suggesting that MMP-9 may not contribute significantly to the pathology [29].

The accumulation of abnormal laminins in the GBM may be more generally applicable to glomerulonephritis. It will be important to determine specifically which abnormal laminin heterotrimers accumulate in the GBM in membranous glomerulonephritis where GBM deposition of laminin β1 has been described [27], whether FAK is activated, and whether elevated glomerular expression of MMPs is observed. Such data would implicate the use of FAK blockade as a potential therapeutic approach for this glomerular disease as well as for Alport syndrome. A recent study using both lipopolysaccharide (LPS) and anti-GBM antibody-induced glomerular disease models showed that podocyte injury could be limited by blocking FAK activation [22], providing further evidence of the potential general utility of FAK inhibitors for the treatment of glomerular diseases. It should be noted, however, that safe FAK inhibitors have yet to be developed. As we mentioned, prolonged administration of TAE226 stunted the growth of treated wild type and Alport mice in our studies, precluding lifespan studies and underscoring the toxicity issues that have prevented development of clinically relevant compounds.

Laminin α2-mediated FAK activation is also observed in the CD151 knockout mouse model (**Figure 3**). Like the Alport mouse, the CD151 knockout mouse shows massive up-regulation of both MMP-10 and MMP-12 (**Figure 4**), providing further evidence that induction of these genes is likely regulated, at least in part, by FAK activation. We included the CD151 knockout mouse in our study because previous work had shown that this mouse model has ultrastructural defects in the GBM that are identical to that observed in Alport mice, and the mature GBM contained laminin β1 chain, which is not present in wild type mature GBM [30]. Furthermore, this model has normal GBM type IV collagen composition, ruling out the possibility that the altered Alport GBM collagen composition (comprised entirely of α1(IV) and α2(IV) chains) might directly activate FAK in podocytes. MMP-10 expression in the glomerulus has not been previously documented, likely owing to its low abundance in healthy glomeruli. Immunostaining for MMP-10 (**Figure 4**B) showed that MMP-10 is undetectable in the wild type glomeruli and abundant in the Alport glomeruli. MMP-10, like its related stromelysin MMP-3, has a broad substrate specificity, which includes type IV collagen [31], [32]. The high levels of induction observed (700- to 1200-fold) suggest that MMP-10 might play an important role in the pathogenic mechanism of Alport glomerular disease, warranting further study.

In earlier work we have shown that biomechanical strain, most likely owing to the change in basement membrane composition, has pro-pathogenic consequences in Alport glomeruli. These include exacerbating GBM destruction by way of MMP induction and accelerating the invasion of glomerular capillaries by mesangial processes [11], [15]. Here we show that biomechanical stretching of podocytes directly activates FAK and induces the expression of MMP-10 and MMP-12 (**Figure 6**). Treatment of stretched cells with the FAK inhibitor TAE226 blocked MMP induction in this same set of experiments. We also showed that stretching podocytes caused nuclear localization of NF-kappaB (**Figure 7**), consistent with its activation [33], and that adding a peptide inhibitor for NF-kappaB to stretched cells blocked the induction of MMP-10. Collectively, these data suggest that biomechanical strain exacerbates laminin α2-mediated activation of FAK in podocytes leading to NF-kappaB-dependent induction of MMP-10 (and likely other pro-inflammatory genes).

In summary, here we have defined a role for GBM laminin α2 in Alport glomerular pathogenesis by way of activation of FAK on glomerular podocytes leading to the downstream activation of MMP-9, MMP-10, and MMP-12 gene expression. It should be noted, however, that there are other mesangial laminins that might contribute to podocyte FAK activation, for example laminin 411 and 421 [34]. This mechanism of MMP induction likely involves NF-kappaB activation and is exacerbated by biomechanical strain on the glomerular capillary tuft. We show that systemic inhibition of FAK by way of a small molecule inhibitor ameliorates both glomerular and tubulointerstitial pathologies in our model, likely owing to its effects not only on glomerular podocytes, but also mesangial cells, and possibly firm adhesion-mediated monocyte activation. Since laminin α2 starts to be deposited in the Alport GBM as early as 10 days in the 129 Sv/J autosomal mouse model, where proteinuria is not detected until 3 weeks of age, we propose that this represents one of the earliest events underlying the development of Alport glomerular disease. This mechanism may be generally applicable to other forms of glomerulonephritis where the accumulation of “abnormal” laminins in the GBM has been documented.

## Supporting Information

Figure S1 FAK activation in both Alport and CD151 knockout glomeruli occurs in the glomerular podocytes. Glomeruli from wild type (7 week 129 Sv), Alport (7 week 129 Sv), and CD151 (10 week FVB) mice were dual immunostained using antibodies specific for pFAK^397^ (red) and the podocyte nuclear marker WT1. Merged images show that the FAK positive cells are also positive for WT1, indicating that FAK activation is occurring in the podocytes.
(TIF)

Figure S2 Further evidence supporting mesangial filopodial invasion of the glomerular capillaries in Alport mice. Immunogold labeling for integrin α8, which is only found expressed in the mesangial cells of the glomerulus, in a “bleb” of an Alport capillary loop (arrow). Note the absence of immunogold labeling in the podocyte pedicles (asterisks) and the fenestrated endothelium (arrowheads).
(TIF)
