# Supplementary figures and images for: Laminin α2-Mediated Focal Adhesion Kinase Activation Triggers Alport Glomerular Pathogenesis
Source: PLoS One. 2014 Jun 10;9(6):e99083. doi: 10.1371/journal.pone.0099083 (PMC4051676; doi:10.1371/journal.pone.0099083)

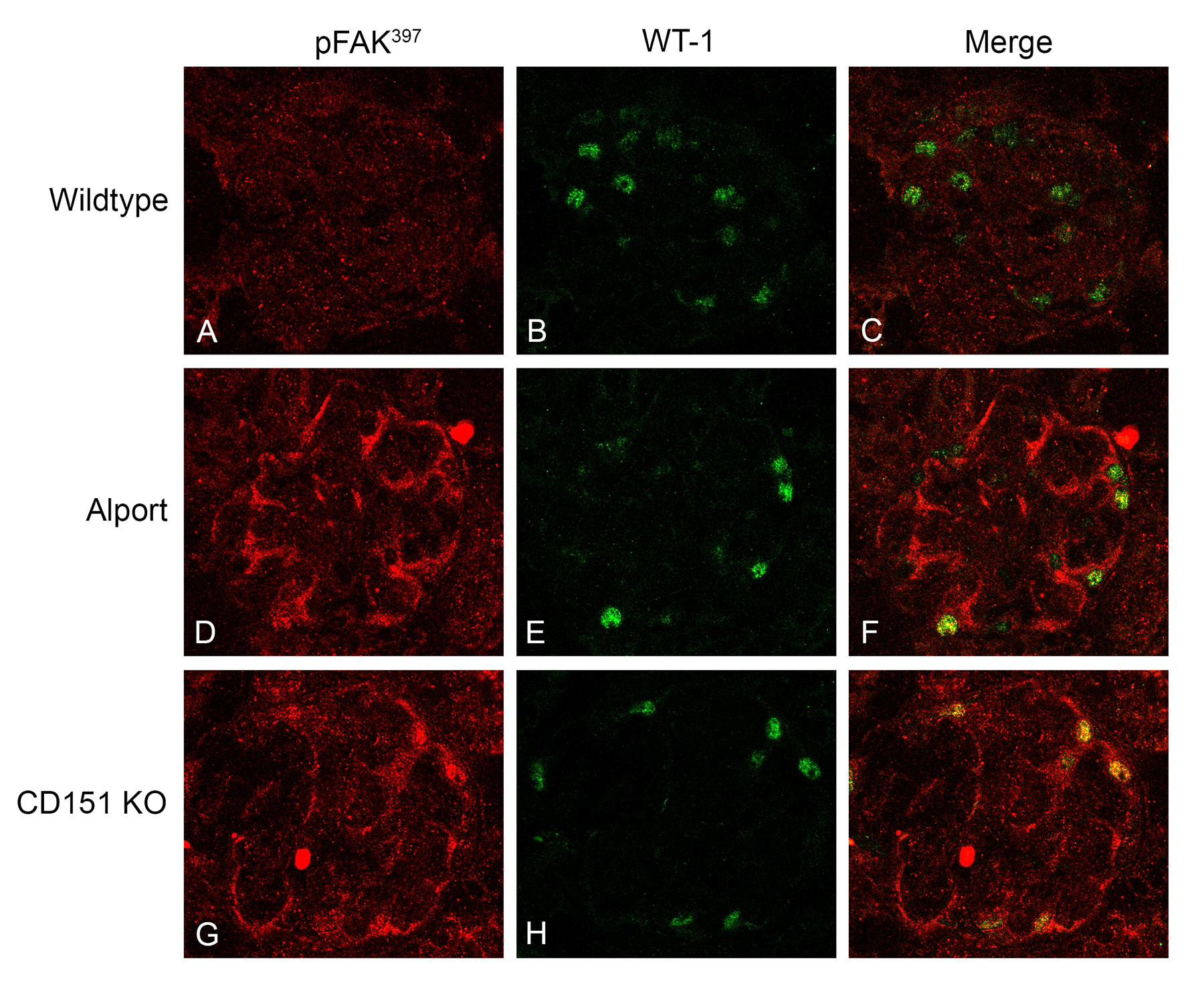

Supplement: Figure S1 — FAK activation in both Alport and CD151 knockout glomeruli occurs in the glomerular podocytes. Glomeruli from wild type (7 week 129 Sv), Alport (7 week 129 Sv), and CD151 (10 week FVB) mice were dual immunostained using antibodies specific for pFAK397 (red) and the podocyte nuclear marker WT1. Merged images show that the FAK positive cells are also positive for WT1, indicating that FAK activation is occurring in the podocytes. (TIF) [file pone.0099083.s001.tif]

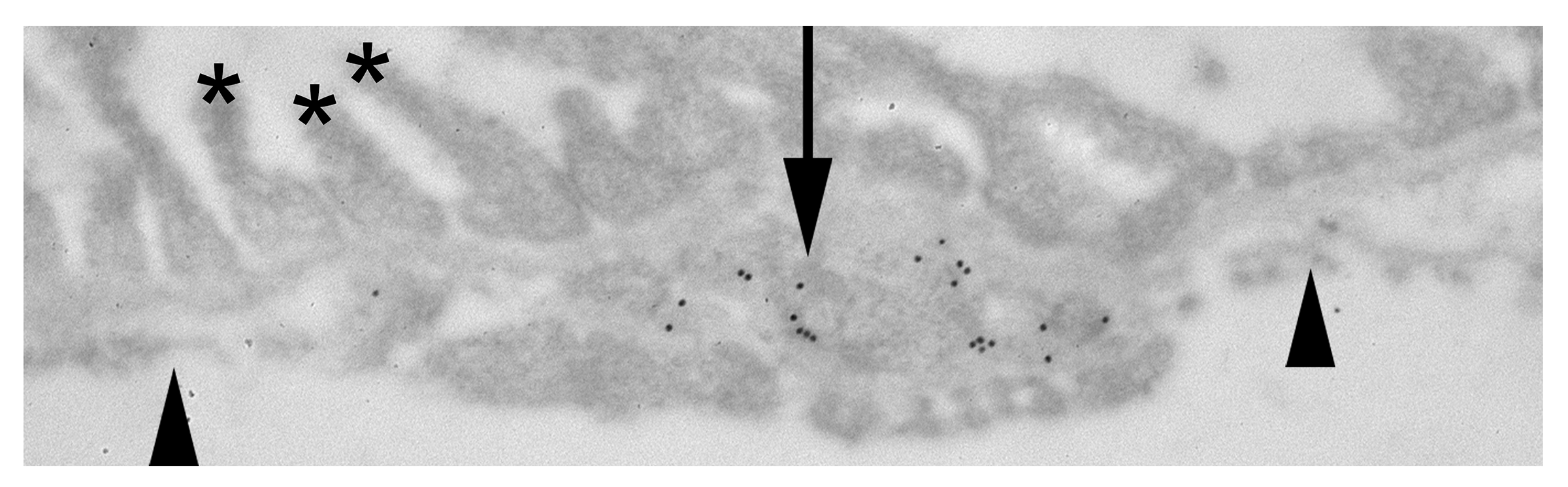

Supplement: Figure S2 — Further evidence supporting mesangial filopodial invasion of the glomerular capillaries in Alport mice. Immunogold labeling for integrin α8, which is only found expressed in the mesangial cells of the glomerulus, in a “bleb” of an Alport capillary loop (arrow). Note the absence of immunogold labeling in the podocyte pedicles (asterisks) and the fenestrated endothelium (arrowheads). (TIF) [file pone.0099083.s002.tif]
